# The Surgical Management of a Giant Innominate Artery Aneurysm in a Patient With Coronary Disease: A Case Report

**DOI:** 10.7759/cureus.13038

**Published:** 2021-01-31

**Authors:** Ramia Bougrine, Hanane Aissaoui, Noha Elouafi, Ihsane Alloubi, Nabila Ismaili

**Affiliations:** 1 Department of Cardiology, Mohammed I University/Mohammed VI University Hospital/Epidemiological Laboratory of Clinical Research and Public Health, Oujda, MAR; 2 Department of Cardiovascular Surgery, Mohammed I University/Mohammed VI University Hospital/Epidemiological Laboratory of Clinical Research and Public Health, Oujda, MAR

**Keywords:** innominate artery aneurysm, coronaropathy, coronary artery bypass

## Abstract

The innominate artery aneurysm (IAA) accounts for a small percentage of all peripheral aneurysms. However, its clinical outcomes are potentially devastating, especially when it is associated with coronary disease, due to the high risk of spontaneous rupture and thromboembolic complications. Surgical repair is always recommended in such cases. The treatment of such a condition presents a surgical challenge with high morbidity and mortality rates. In this report, we discuss the case of a 56-year-old male who presented with a right cervical mass secondary to a large IAA with underlying coronary artery disease. The patient underwent a simultaneous operation for IAA and coronary bypass grafting.

## Introduction

An innominate artery aneurysm (IAA) is a rare condition. However, it can be potentially severe due to the associated high risk of complications, even in asymptomatic patients. Surgery remains the gold standard to prevent rupture, compression, and thromboembolic risks in IAA patients. Even though the open repair is an effective treatment method, it is associated with significant morbidity and mortality rates, especially when combined with coronary artery disease requiring more invasive and complex surgical procedures. Approximately 4% of all innominate artery surgeries tend to be for aneurysmal diseases [1]. The IAA is usually detected as an asymptomatic mass, or it may present itself immediately with neurological complications [2] or even a cardiac arrest due to an aneurysm rupture. We describe our experience with a giant thrombosed IAA in a patient with severe coronary artery disease.

## Case presentation

**Figure 1:**
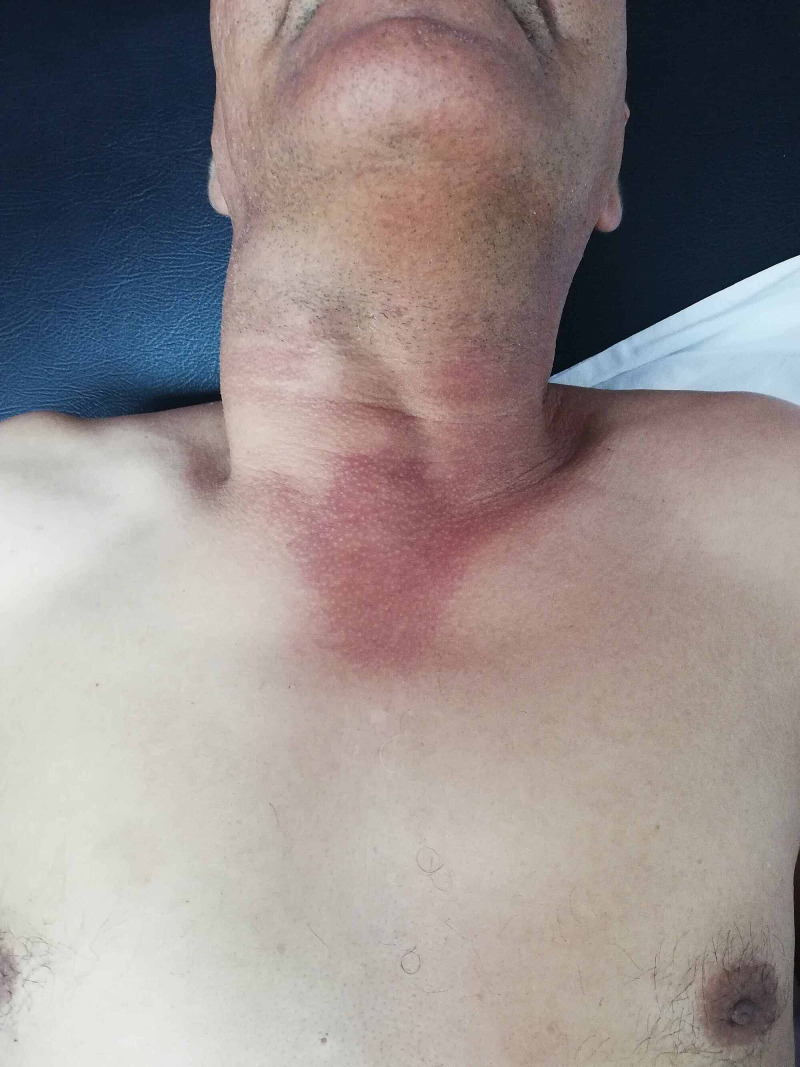
Image of the big mass on the right neck of the patient

**Figure 2:**
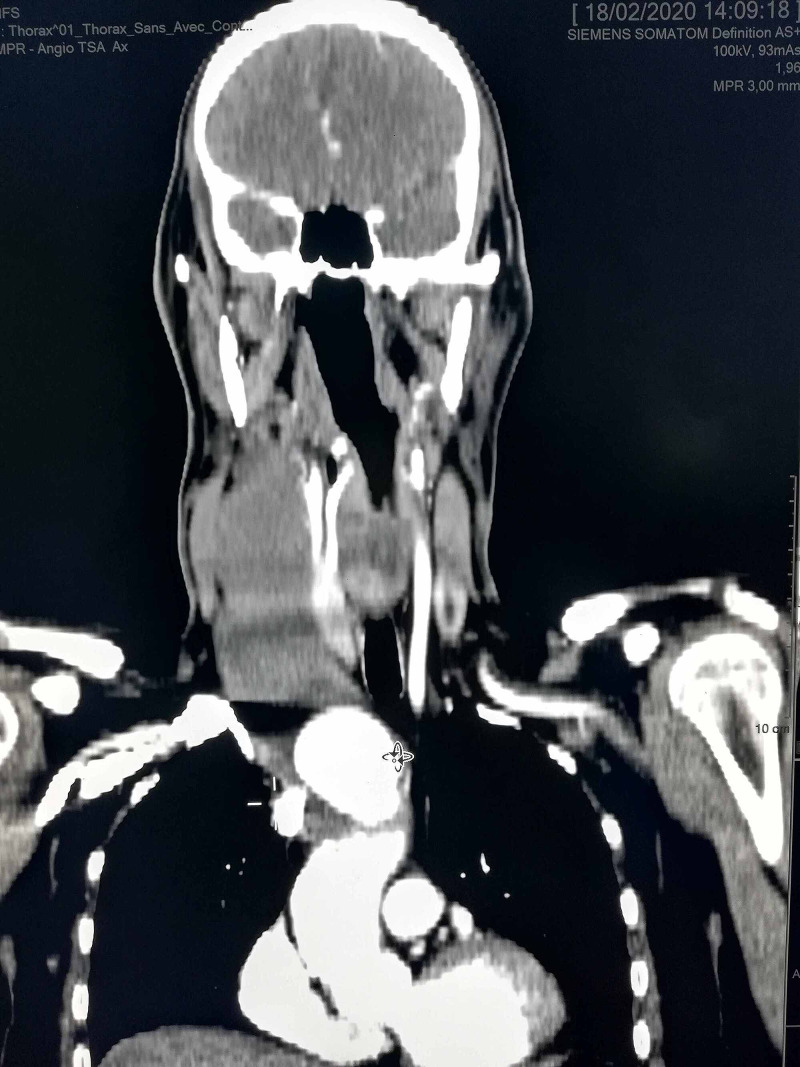
The large thrombosis innominate artery aneurysm is seen at the thoracic TDM

**Figure 3:**
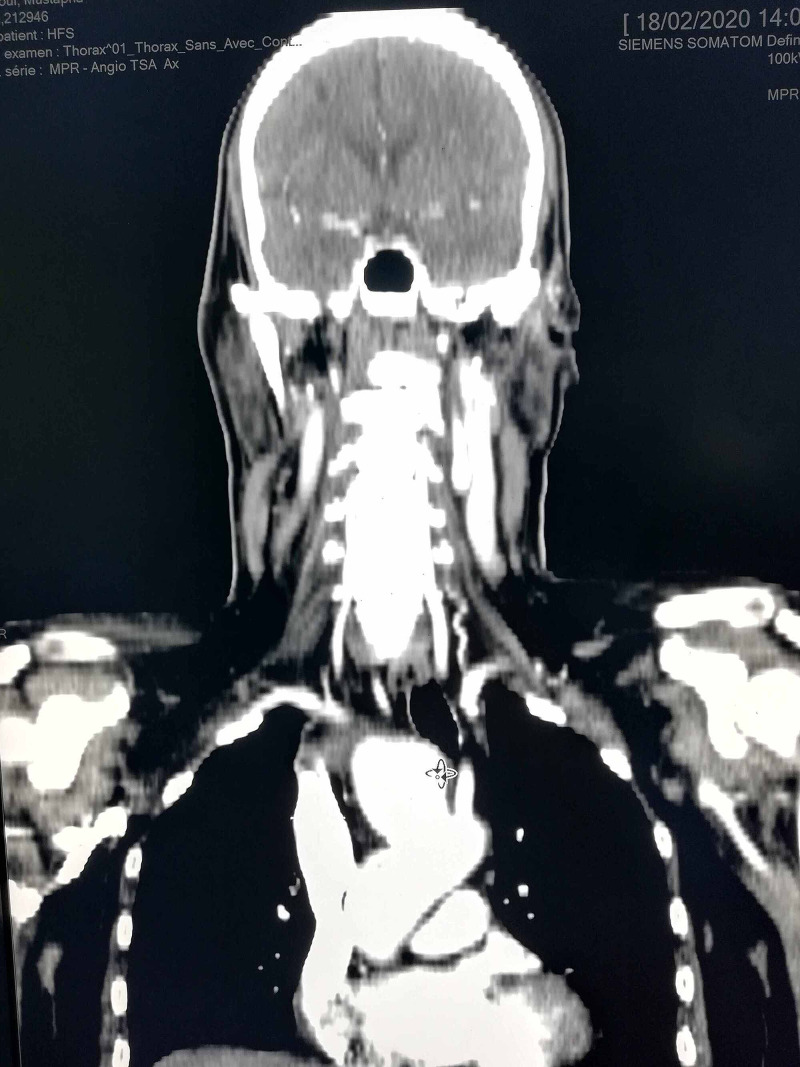
Innominate artery aneurysm not involving the origin of right carotid and subclavian artery

**Figure 4:**
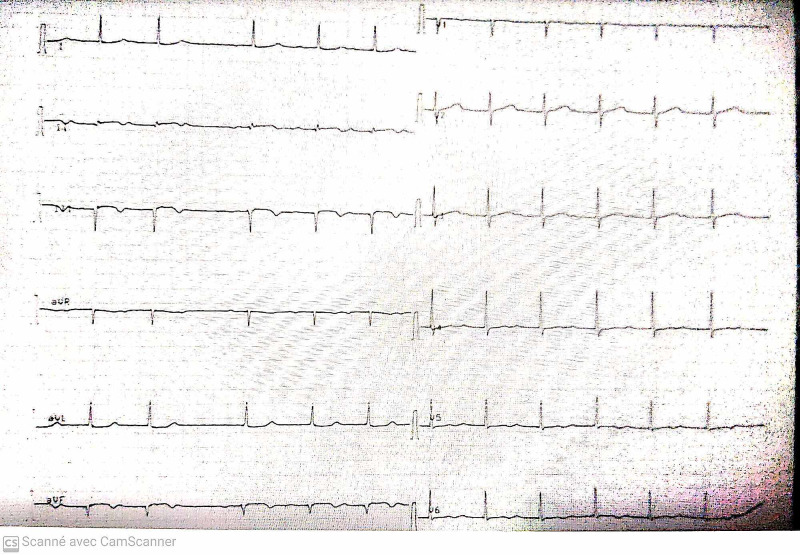
Electrocardiogram of the patient

**Figure 5:**
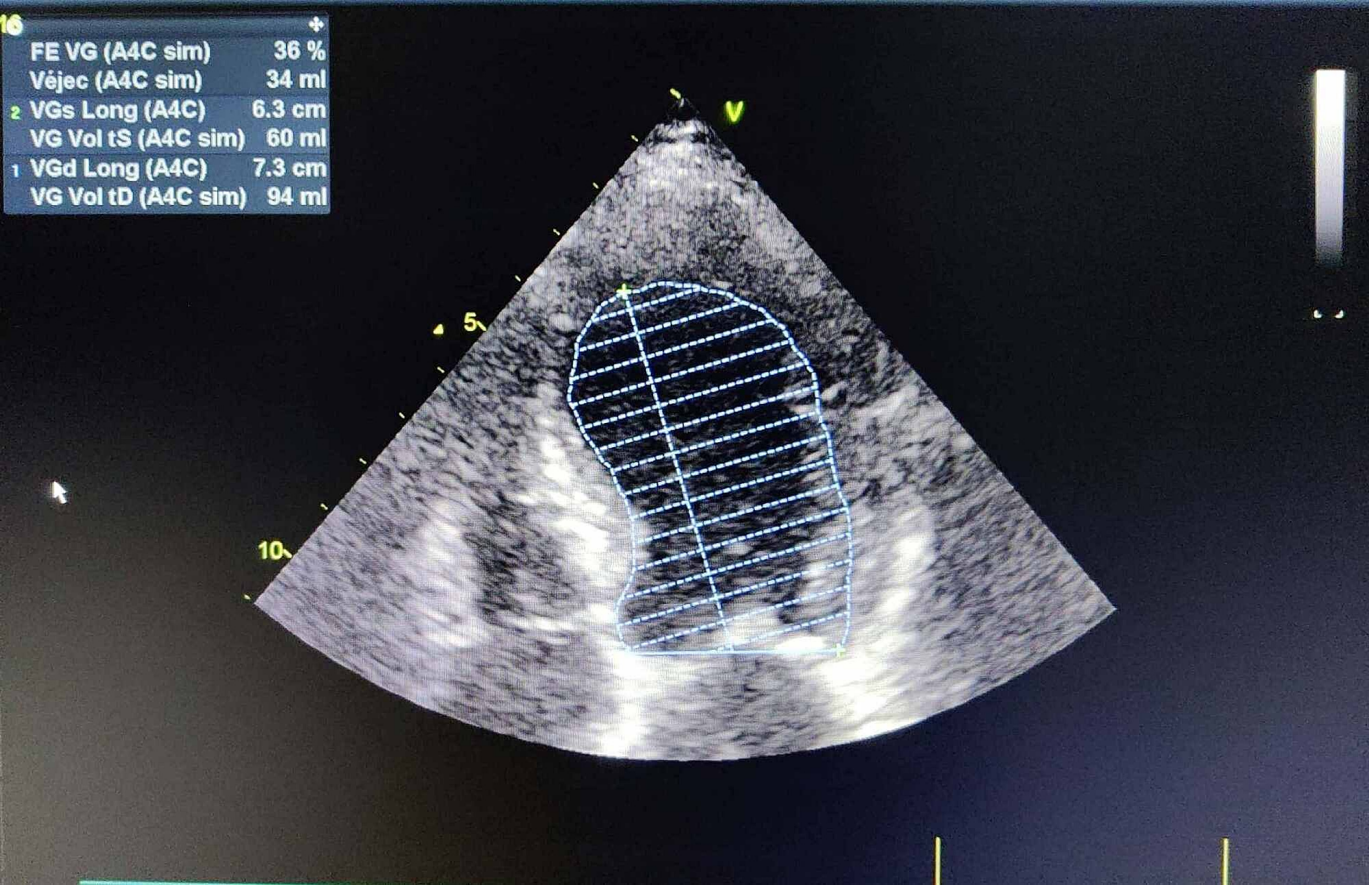
Echocardiography showed a severe VG dysfonction

A 56-year-old male was admitted to our hospital for an increasing swelling mass at the root of his right neck (Figure 1). He had an active smoking history, but no history of chest pain or trauma. Physical examination found a large pulsating mass on the right neck, without other significant clinical findings. Angio-CT scan revealed a large thrombosed aneurysm of 65 x 60 x 170 mm, originating from the innominate trunk; there was no involvement of the origin of the right carotid and subclavian arteries (Figure 2, Figure 3). The electrocardiogram revealed a previous inferior wall myocardial infarction with negative T-waves in the inferior leads (Figure 4). Transthoracic echocardiography showed severe depressed left ventricular function [ejection fraction (EF): 35%] (Figure 5). Coronary angiography revealed atherosclerotic coronary disease with chronic occlusion of the right proximal coronary artery (RCA), severe stenosis of the main of the diagonal artery, and severe stenosis of the circumflex artery. Myocardial viability study confirmed the presence of viable myocardium in the lateral wall and the anterior wall with non-viable myocardium of the inferior wall. A CT angiogram scan of the cerebral arteries showed a permeable Willis polygon.

The decision was made to perform a simultaneous operation of the IAA resection and coronary artery bypass grafting (CABG).

**Figure 6:**
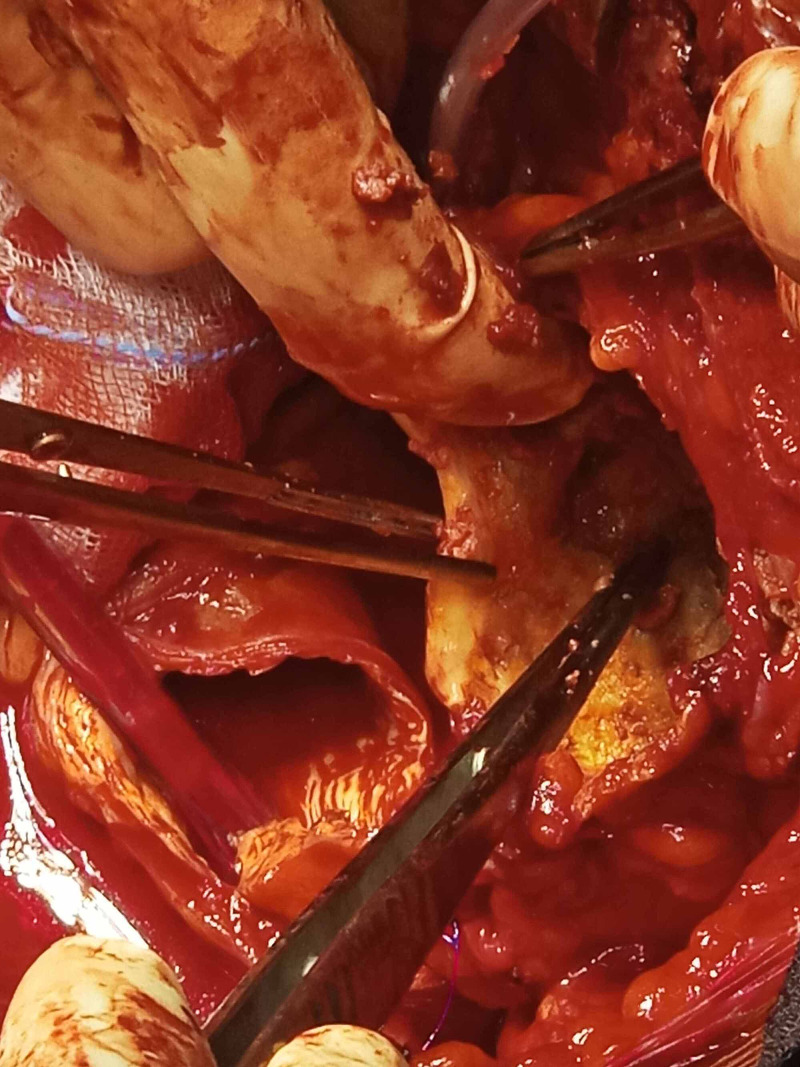
Intraoperative image of the innominate artery aneurysm

Under general anesthesia, peripheral femoral arterial cannulation was performed along with the catheterization of the left carotid artery; a median sternotomy extending into the right side of the neck along with the medial edge of the sternocleidomastoid muscle was performed. Cardiopulmonary bypass (CPB) circuitry was completed with cannulation of the superior and inferior vena cava, and the patient was cooled to 25 °C. The myocardial arrest was achieved by cold crystalloid cardioplegia. The fragile part of the ascending aorta was clamped and resected over the coronary ostia at the level of the sinotubular junction (Figure 6).

**Figure 7:**
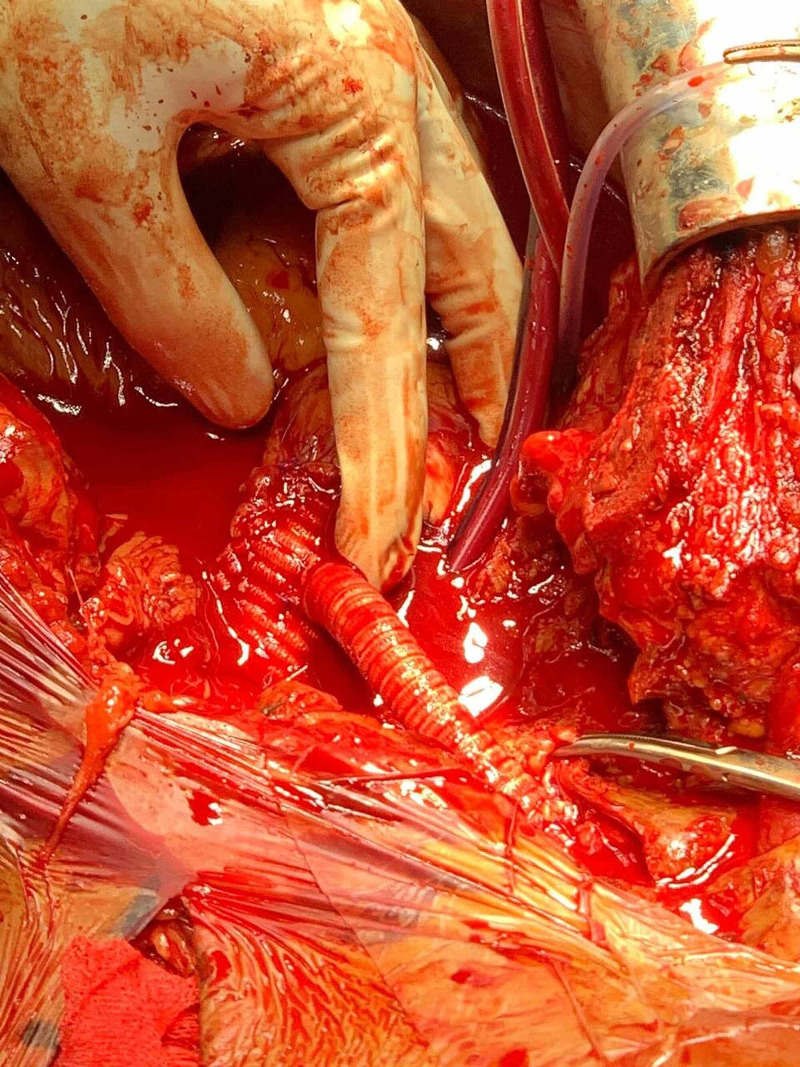
Aortic Dacron graft with an innominate prosthetic graft

A Dacron aortic graft was anastomosed proximally to the aortic sinotubular junction with external reinforcement by a Teflon strip. CPB flow was stopped. The IAA was completely resected from its origin in the arch to its distal bifurcation. The Dacron graft was inserted and the distal lateral ends ligated. The distal end of the graft was anastomosed to the proximal aortic arch and reinforced with external Teflon bands. The other end of the graft was anastomosed to the distal innominate artery (Figure 7).

Unilateral anterograde selective cerebral perfusion was instituted via a cerebral perfusion catheter placed into the left common carotid artery. Cerebral monitoring was achieved using transcutaneous cerebral oximetry.

We proceeded with the coronary bypass with retrograde cardioplegia, with a circulatory arrest time of 42 minutes. Resumption with reheating for the preparation for aortic unclamping was disastrous with myocardial fibrillation not recoverable after electric shock or with supplemental doses of cardiotonic drugs. Without the possibility of a heart transplant immediately, we declared the patient’s death.

## Discussion

An IAA is usually an asymptomatic mass. When identified, an IAA requires surgical management to prevent complications. This case report aimed to discuss the dilemma of combined surgery of an aortic aneurysmal pathology associated with bypass surgery: is it feasible to combine two extremely complicated procedures or was it necessary to separate them to prevent the fatal outcome in our case?

Anatomical classifications of the IAA have been proposed by Kieffer et al. [3] and includes three groups: (1) group A: rare, the origin of the IA is not involved; (2) group B: the most common one; it does not involve the aorta but involves the origin of the IA; and (3) group C: involves both the IA and the ascending aorta.

Studies have shown that IAA is mainly caused by atherosclerosis; however, other causes may include vasculitis, either infectious like syphilis or tuberculosis [4-5]. A study reported that among 120 patients with IAA, the etiology was unknown in 58.3% [6-7]. IAA represents 3% of supra-aortic vessel aneurysms and can lead to devastating complications [8]. The rupture rate is 11% in these patients, especially when the diameter exceeds 3 cm, in which case the risk of rupture will be higher [3-9].

Kieffer et al. [3] have proposed that asymptomatic patients with isolated IAA, with a saccular form or transverse diameter of >3 cm, should undergo surgery. Various surgical approaches have been tried and they include ligation alone [10], patch angioplasty [11], resection with end-to-end anastomosis [12], and bypass. The treatment choice remains a subject that is still debated, especially in ‘high-risk’ patients with complex anatomy and with preexisting comorbidities like in our case. These factors increase the risk of surgical complications and mortality [13]. Surgical repair is the standard approach to IAA. Endovascular treatment is also limited by the IAA classifications [6].

Generally, open surgical repair is performed via a median sternotomy, including CPB and induced hypothermia. Although this treatment offers excellent long-term results [14-15], additional bypass surgery poses a considerable high surgical risk, which results in extremely high operative mortality rates compared to an IAA surgery alone. In the present case, we reported the fatal outcome of a simultaneous surgery of IAA and coronary artery bypass due to severe myocardial failure.

Multiples series have reported a simultaneous operative approach toward an aortic aneurysm along with coronary bypass [16-19], with significant mortality rates between 14-55% [16]. The most frequent adverse effects have been perioperative myocardial failures, permanent cerebral dysfunctions, and massive intraoperative hemorrhages [17]. We looked into the reasons why CABG increases operative mortality and discussed the ways to improve the results in these cases.

Ehrlich et al. analyzed 271 cases of aortic aneurysms with hypothermic cardiac arrest to determine the variables associated with an adverse outcome [17]. They found causes related to coronary artery bypass surgery, cerebral ischemic time, and prolonged intubation [17].

Yamashiro et al. analyzed 24 patients who underwent simultaneous surgery replacement of aortic aneurysm and CABG. There was no significant difference in either survival or the cardiac-event-free rate at five years [18]. They concluded that thoracic aortic replacement can be performed safely even if is associated with CAP and that coronary artery revascularization is important to prevent cardiac events [18].

A less invasive procedure can be performed before this higher risk procedure, A complete coronary revascularization with angioplasty can prevent perioperative acute myocardial failure; Ueda et al. have reported that incomplete coronary revascularization with significant stenosis represents a significant risk factor for perioperative cardiac events in the aortic aneurysm cases associated with coronary artery disease [19]. Revascularization is recommended if there are a major coronary artery and proximal stenosis (>75%). Therefore, percutaneous coronary intervention (PCI) may be the choice of coronary revascularization before proceeding to an aneurysmal corrective surgical procedure. Besides, we have another challenge that arises with respect to antiplatelet therapy, which increases the risk of bleeding during the following surgery [20]. Cases like this throw up a management issue that requires a multidisciplinary approach and an experienced technical platform [20].

## Conclusions

The brachiocephalic trunk can be the site of an aneurysmal pathology with serious complications, especially in cases with significant coronary artery disease and severely depressed left ventricular function, A simultaneous surgical approach should be specifically considered. Repair of IAA in patients without comorbidities is recommended and can be performed with low mortality and morbidity with favorable long-term survival.

Our recent experience was unique and presented a management challenge; we recommend a decrease in cardiac ischemic time and cardiopulmonary time, and if possible, complete revascularization should be achieved before the repair of IAA to improve operative morbidity and mortality.
